# New strategy of color and power doppler sonography combined with DMSA in the assessment of acute pyelonephritis in infants

**DOI:** 10.1186/s12882-021-02390-2

**Published:** 2021-05-17

**Authors:** Min Guang Chen, Yan Yang, Qing Yang, Jie Qiu Zhuang, Xiao Hua Ye, Wen Jie Zheng

**Affiliations:** 1 Department of Nephrology, The Second Affiliated Hospital and Yuying Children’s Hospital of Wenzhou Medical University, Wenzhou, 325027 Zhejiang Province China; 2 Department of Ultrasonics, The Second Affiliated Hospital and Yuying Children’s Hospital of Wenzhou Medical University, Wenzhou, 325027 Zhejiang Province China; 3 Department of Rheumatology, The Second Affiliated Hospital and Yuying Children’s Hospital of Wenzhou Medical University, Wenzhou, 325027 Zhejiang Province China

**Keywords:** Color and power doppler sonography, 99mTc-dimercaptosuccinic acid scintigraphy, Acute pyelonephritis, Children, Kidney

## Abstract

**Background:**

The purpose of this study was to evaluate the clinical value of color and power doppler sonography (CPDS) when combined it with 99mTc-dimercaptosuccinic acid scintigraphy (DMSA) in assessment of acute pyelonephritis (APN) in infants.

**Methods:**

A total of 79 children with APN admitted to our hospital from June 2016 to Jan 2019 were enrolled, including 52 boys and 27 girls, age range 1 month to 3 years old. All cases followed the diagnostic criteria for acute pyelonephritis and excluded anatomical abnormalities of urinary system. All 79 patients were examined by urinary ultrasonography (US), CPDS, and DMSA within 48 h of fever and analyzed the clinical value of combining the two methods in the assessment of APN in infants.

**Results:**

Among 79 children, urinary ultrasonography revealed 2 cases of renal cortical echo changes, both located in the upper pole of the kidney, 24 cases of kidney enlargement, and 1 case of left kidney shrinkage. Ninety-five kidneys were shown to be diseased with DMSA, while 105 kidneys abnormal by CPDS. The sensitivity of CPDS was 69.4%, and the specificity was 38.1%. In children younger than 6 months, the sensitivity of CPDS was 56.9%, which was 84.2% in childeren between 6 months to 1 year, and 94.4% from 1 to 3 years old, respectively. The corresponding specificity of CPDS was 44.1, 26.7, and 35.7%. There was no significant correlation between CPDS levels and DMSA positive results. The abnormal rate of intermediate part in the kidneys was significantly lower than that in the upper and lower poles. Children with abnormal CPDS have a greater risk of renal scarring(*p* < 0.05).

**Conclusion:**

Abnormalities detected by CPDS in a cohort of infants with APN poorly correlated with DMSA findings. But the sensitivity of CPDS is highly age-related, it can be used as a non-invasive helpful tool for early diagnosis of acute pyelonephritis in infants older than 6 months old.

## Background

Urinary tract infection(UTI) is a common disease in children. Although most of the patients with good prognosis, acute pyelonephritis (APN), if not be treated promptly, can lead to permanent renal scar formation, which is an important cause of chronic renal failure in young people. Therefore, it’s important to identify APN early and give it positive treatment [1]. However, the accurate diagnosis of APN in children still quite difficult on the basis of clinical and laboratory findings alone [2, 3].

Since 1972, The technetium 99 m dimercaptosuccinic acid (DMSA) has been considered to be a gold standard for diagnosing APN at present [4]. However, there are still many disadvantages. Quite a few hospitals do not have this inspection equipment which is radiation and expensive. Therefore, a simple, inexpensive and non-radiative alternative method is required to help early identification and follow-up of children with APN.

Previous studies have shown that color and power doppler sonography (CPDS) can be used to evaluate renal cortical blood flow and contribute to the diagnosis of APN [5, 6], and compared the diagnostic difference between CPDS and DMSA in children with APN [7–12]. Some studies believe that doppler ultrasonography can be used as a predictive tool for permanent kidney damage following acute pyelonephritis [13],while others disagree [14, 15].

Most of the above studies focused on whether the CPDS could instead of DMSA for the diagnosis and follow-up of APN. But in our opinion, because CPDS can provide information such as renal cortical blood flow that DMSA and US cannot provide in the assessment of APN, negative DMSA sometimes cannot completely rule out APN [7]. Therefore, it may be better to make full use of the two inspections’ respective advantages instead of replacing them. Thus, the purpose of this study was to assess the new role of CPDS in the application of APN in infants by prospective combined use of DMSA.

## Mathods

### Study population

We enrolled 79 children (including 52 boys and 27 girls) diagnosed with acute pyelonephritis, who were admitted to the Yuying Children’s Hospital of Wenzhou Medical University from June 2016 to January 2019. All the patients were between 1 month and 3 years old. They all met the diagnosis criteria of APN [16], including positive urine culture or pyuria, body temperature above 38.5 °C, and raised peripheral blood leukocytes or C-reactive protein(CRP). Congenital malformations of the urinary system were excluded.

### Data collection

All 79 patients underwent CPDS and DMSA examinations within 48 h of hospitalization. In order to ensure the accuracy of the test results, the CPDS of all children were individually checked by two senior sonographers who did not know the results of the DMSA test in advance. We operated the Siemens Sequoia 512 scanner for inspection. Ultrasonic probe frequency was 8–14 MHz for children under 3 months and 2.5–4 MHz for children older than 3 months. An empirical 9-point semi-quantitative analysis was used as previously published [16]. The kidney was artificially divided into the upper pole, middle pole, and lower pole, and the parenchymal perfusion in each area was scored. No perfusion was scored as 0 and normal perfusion was 3 points. The sum of the three regions scores was the total score of each kidney. An 8–9 score was regarded CPDS negative,meanwhile, the score of CPDS positive was defined as less than eight.

The 99mTc-DMSA examination was performed by a senior doctor of ECT who was unaware of the results of CPDS. We used SPECT vertex v60 ADAC (USA) for Tc-99 m DMSA renal scintigraphy. Each child was injected intravenously with 3.7 MBq/kg (0.1 mCi/kg) Tc-99 m DMSA, Orbiter Siemens gamma camera was used to obtain images with 300,000–500,000 counts after 2–4 h. A same 9-point method for semi-quantitative analysis was selected which also divide the kidney into three regions of the upper pole, middle pole, and lower pole. No radioactive uptake was scored as 0 and normal uptake was 3 points. Greater than or equal to 8 points was considered DMSA negative, and a score of less than 8 was DMSA positive [17].

### Statistical analysis

Comparisons between CPDS or DMSA findings were performed using Mann-Whitney nonparametric tests. The diagnostic values (sensitivity, specificity, predictive values, and accuracy) of CPDS and DMSA were assessed with contingency Tables. A *P* value ≤0.05 was considered significant.

## Results

### Comparison of US, CPDS, and DMSA results in children

**Fig. 1:**
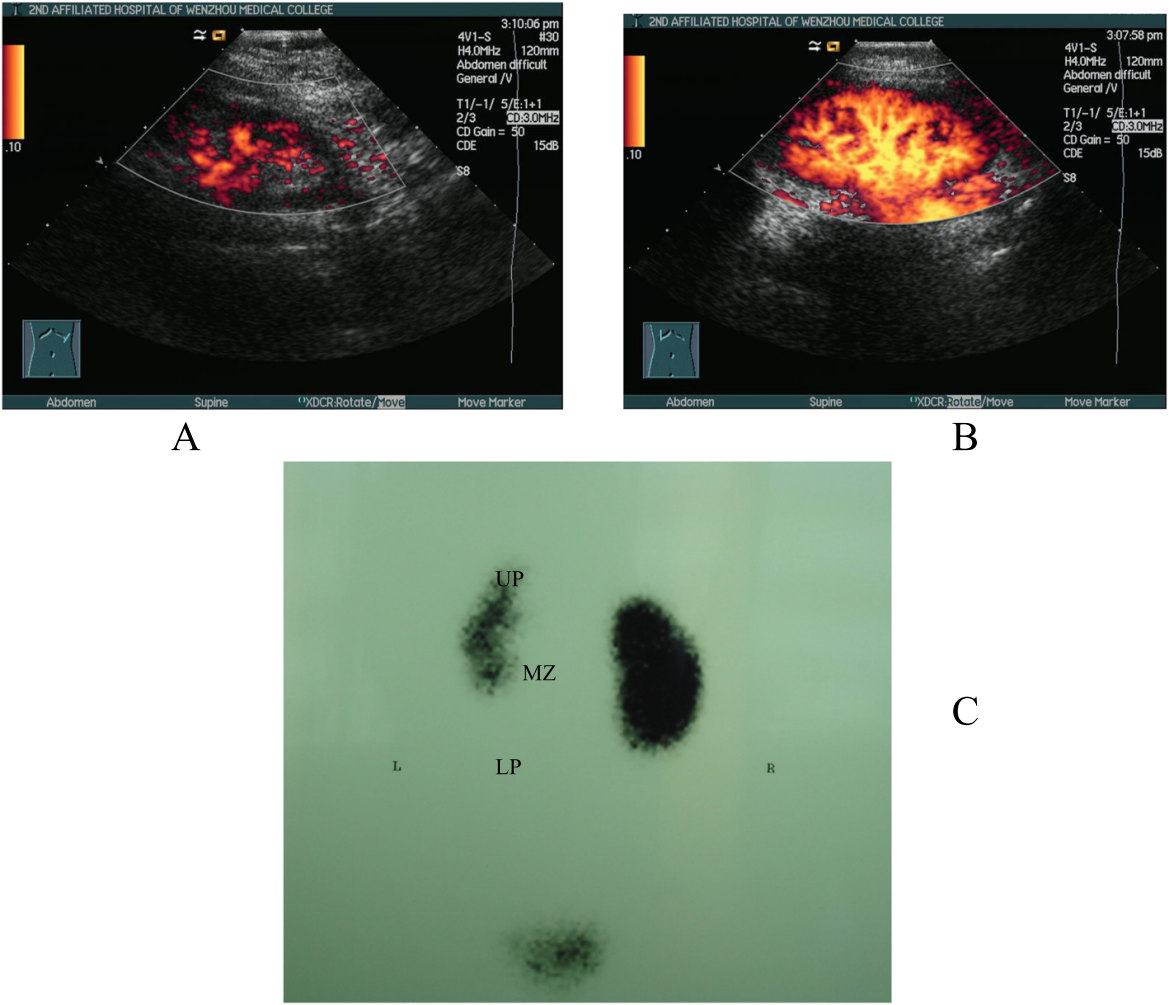
Comparison of CPDS and DMSA results in infants with acute pyelonephritis. **a**, CPDS result of the left kidney, renal cortical blood flow was significantly decreased; **b**, CPDS result of the right kidney, renal cortical blood flow was normal; **c**, DMSA result, the upper pole, midzone, and lower pole of the left kidney were significantly sparse, but the right kidney was normal. UP, upper pole; MZ, midzone; LP, lower pole

All 79 patients met the APN diagnostic criteria. Urinary ultrasonography revealed 2 cases of renal cortical echo changes which both located in the upper pole of the kidney, 24 cases (48 kidneys) of kidney enlargement, and 1 case of left kidney shrinkage. For patients with APN, CPDS and DMSA can provide clear and valuable imaging results from different angles (see Fig. 1). Among 79 children (158 kidneys), 95 kidneys had abnormal DMSA and 105 had abnormal CPDS. The gross US changes were more apparent in the DMSA+ group (x^2^ = 19.397, *P* < 0.01) (See Table 1). The sensitivity, specificity, positive predictive value, and negative predictive value of CPDS as follow. (See Table 2).

**Table 1:** Comparison of US and DMSA results in children

Group	US +	US -	Total
DMSA +	42	53	95
DMSA -	7	56	63
Total	49	109	158

*+* Positive, − Negative

**Table 2:** Comparison of CPDS and DMSA results in children

Group	DMSA +	DMSA -	Total
CPDS +	66(SEN 69.4%, PPV 62.8%)	39	105
CPDS -	29	24(SPE 38.1%, NPV 45.3%)	53
Total	95	63	158

*+* Positive, *−* Negative, *SEN* Sensitivity, *SPE* Specificity, *PPV* Positive predictive value, *NPV* Negative predictive value

### Comparison of CPDS and DMSA results in children with different age

The sensitivity of CPDS detection was lower in children younger than 6 months of age, and the sensitivity increased with age. The sensitivity, specificity, positive predictive value, and negative predictive value of CPDS in different groups as follow. (See Table 3).

**Table 3:** Comparison of CPDS and DMSA results in children of different age groups (B)

Group	Group 1(< 6 M)			Group 2(6 M ~ <1Y)			Group 3(1Y ~ <3Y)		
Group	DMSA +	DMSA -	Total	DMSA +	DMSA -	Total	DMSA +	DMSA -	Total
CPDS +	33	19	52	16	11	27	17	9	26
	(SEN 56.9%; PPV 63.4%)			(SEN 84.2%; PPV 59.2%)			(SEN 94.4%; PPV 65.4)		
CPDS -	25	15	40	3	4	7	1	5	6
	(SPE 44.1%; NPV 37.5%)			(SPE 26.7%; NPV 57.1%)			(SPE 35.7%; NPV 83.3%)		
Total	58	34	92	19	15	34	18	14	32

*+* Positive, *−* Negative, *SEN* Sensitivity, *SPE* Specificity, *PPV* Positive predictive value, *NPV* Negative predictive value

### Comparison of abnormal results of different kidney zones

There was no significant correlation between CPDS levels and DMSA positive results(*P >* 0.05). No matter CPDS or DMSA, the abnormal rate in midzone of kidney was significantly lower than that in the upper and lower poles (See Table 4,).

**Table 4:** Comparison of results of different kidney sites

	Upper pole			Midzone			Lower pole		
groups	DMSA +	DMSA -	Total	DMSA +	DMSA -	Total	DMSA +	DMSA -	Total
CPDS +	43	35	78	6	21	27	31	39	70
CPDS -	28	52	80	12	119	131	31	57	88
Total	71	87	158	18	140	158	62	96	158

## Discussion

APN is considered to be one of the most common and serious illnesses in infants. It may lead to permanent renal scar formation if it does not be recognized and be used by effective antibiotic treatment at the first 48 h after onset of the disease. A study has shown that the incidence of renal scar even up to 64% of affected pediatric kidneys [18]. Therefore, it is important to assess APN and treat it effectively as early as possible.

Imaging plays an essential role in the diagnosis of APN. Our data showed that a few children had normal white blood cells or normal CRP, but they had fever and abnormal urine tests. Therefore, it is crucial to identify APN by combining with imaging tests.

Tc-99 m DMSA renal scintigraphy has been used for the detection and localization of APN inflammation with high sensitivity and specificity. And it is considered the gold standard for the diagnosis of APN. The invasiveness procedure, radiation exposure, and cost limit its clinical application. Meanwhile, negative DMSA results cannot completely rule out APN [7] . In this study, 7 patients had typical clinical manifestations of APN whose urine cultures were positive, peripheral blood leukocytes and CRP were significantly increased but had negative DMSA. Interestingly, 6 of these 7 cases showed positive CDPS. We diagnosed as APN based on clinical and CPDS and formulated corresponding treatment strategies in time for these patients. However, so far, early diagnosis of APN is still tricky in clinical practice.

Various types of renal vascular and renal parenchymal lesions are closely related to changes in intrarenal arterial hemodynamics. CPDS is developed on color Doppler technology and is superior to color doppler flow imaging in showing sensitivity and continuity of blood flow. Because this test is more sensitive to low-speed blood flow, it reflects the integrated power from the reflected echo of the renal parenchyma [9]. Bude et al. [19] first reported the imaging ability of PDS on renal cortical blood perfusion. Since then, many studies have used PDS to study the effects of various kidney diseases on renal blood flow [15, 20–22]. Previous studies have shown that CPDS can contributes to the diagnosis of APN, Anne Hitzel [7] has shown that although the predictive value of CPDS for renal scar is not high, the results of CPDS and DMSA are consistent in 81% of APN kidneys, suggesting that it has certain clinical application value. In view of the fact that the study did not cover all children and did not involve the exploration of specific parts of the infection, it is necessary to further explore.

Our study showed that the positive rate of DMSA is only 6% in patients older than 6 months with negative CPDS. Two double-blind controlled studies have also suggested that the specificity of CPDS for the diagnosis of APN is 85 to 95%, and the sensitivity is 55 to 75%, which is highly consistent with DMSA examination [5, 8]. The data showed that the sensitive of CPDS is highly age-related. With the increase of age, the sensitivity of CPDS gradually increased. It may be related to a change in kidney blood flow caused by infant crying in an unsedated situation. In order to avoid deviations in the test results, infants should be tested in a quiet state.

Our study also found that there was no significant correlation between CPDS abnormal level and DMSA positive results, which was inconsistent with our pre-expected results. We also observed that the location of renal abnormalities found by CPDS and DMSA is not the same. The pathophysiologic mechanism responsible for CPDS imaging abnormalities are focal ischemia due to vascular compression induced by interstitial edema [23]. While the uptake of 99mTc-DMSA by the renal parenchyma is dependent on the glomerular perfusion and the transport function of the proximal tubule cell membrane. Since the detection mechanism of CPDS and DMSA is different when which be used to detect APN. DMSA abnormalities can not be judged from blood supply abnormalities or renal tubular epithelial cell damage, while CPDS can determine the blood supply of the kidneys. Just because CPDS can provide information such as renal cortical blood flow that DMSA and US cannot provide in the assessment of APN, these two methods have good complementarity.

## Conclusion

It is not necessary to focus on replacing DMSA with CPDS in clinical practice, but to synergistically utilize their respective advantages to improve the clinical APN assessment level. The CPDS can be used as a non-invasive helpful tool for early diagnosis of acute pyelonephritis in infants older than 6 months old, especially when some families refuse DMSA inspection due to radiation exposure.

## Data Availability

The datasets used and/or analyzed during the current study are available from the corresponding author on reasonable request.

## Abbreviations

CPDS: Color and power doppler sonography
DMSA: 99mTc-dimercaptosuccinic acid scintigraphy;
APN: Acute pyelonephritis
US: Urinary ultrasonography
